# Neutrophil–Hepatic Stellate Cell Interactions Promote Fibrosis in Experimental Steatohepatitis

**DOI:** 10.1016/j.jcmgh.2018.01.003

**Published:** 2018-01-08

**Authors:** Zhou Zhou, Ming-Jiang Xu, Yan Cai, Wei Wang, Joy X. Jiang, Zoltan V. Varga, Dechun Feng, Pal Pacher, George Kunos, Natalie J. Torok, Bin Gao

**Affiliations:** 1 Laboratory of Liver Diseases, National Institute on Alcohol Abuse and Alcoholism, National Institutes of Health, Bethesda, Maryland; 3 Laboratory of Cardiovascular Physiology and Tissue Injury, National Institute on Alcohol Abuse and Alcoholism, National Institutes of Health, Bethesda, Maryland; 4 Laboratory of Physiologic Studies, National Institute on Alcohol Abuse and Alcoholism, National Institutes of Health, Bethesda, Maryland; 2 Department of Internal Medicine, Division of Gastroenterology and Hepatology, University of California Davis Medical Center, Davis, California

**Keywords:** Alcohol, High-Fat Diet, Fatty Liver, Reactive Oxygen Species, Inflammation, ALT, alanine aminotransferase, AST, aspartate aminotransferase, cDNA, complementary DNA, *Csf*, colony-stimulating factor gene, CXCL1, chemokine (C-X-C motif) ligand 1, FBS, fetal bovine serum, 4-HNE, 4-hydroxynonenal, G-CSF, granulocyte colony-stimulating factor, GM-CSF, granulocyte-macrophage colony-stimulating factor, IL, interleukin, HFD, high-fat diet, HFD+mB, high-fat diet plus multiple binges, HFD+1B, high-fat diet feeding plus 1 binge of ethanol, HSC, hepatic stellate cell, ICAM-1, intercellular adhesion molecule-1, KO, knockout, MPO, myeloperoxidase, mRNA, messenger RNA, PCR, polymerase chain reaction, RT-PCR, reverse-transcription polymerase chain reaction, ROS, reactive oxygen species, TUNEL, terminal deoxynucleotidyl transferase–mediated deoxyuridine triphosphate nick-end labeling, WT, wild-type

## Abstract

**Background & Aims:**

Hepatic infiltration of neutrophils is a hallmark of steatohepatitis; however, the role of neutrophils in the progression of steatohepatitis remains unknown.

**Methods:**

A clinically relevant mouse model of steatohepatitis induced by high-fat diet (HFD) plus binge ethanol feeding was used. Liver fibrosis was examined. *In vitro* cell culture was used to analyze the interaction of hepatic stellate cells (HSCs) and neutrophils.

**Results:**

HFD plus one binge ethanol (HFD+1B) feeding induced significant hepatic neutrophil infiltration, liver injury, and fibrosis. HFD plus multiple binges of ethanol (HFD+mB) caused more pronounced liver fibrosis. Microarray analyses showed that the most highly activated signaling pathway in this HFD+1B model was related to liver fibrosis and HSC activation. Blockade of chemokine (C-X-C motif) ligand 1 or intercellular adhesion molecule-1 expression reduced hepatic neutrophil infiltration and ameliorated liver injury and fibrosis. Disruption of the *p47*^*phox*^ gene (also called *neutrophil cytosolic factor 1*), a critical component of reactive oxygen species producing nicotinamide adenine dinucleotide phosphate-oxidase in neutrophils, diminished HFD+1B–induced liver injury and fibrosis. Co-culture of HSCs with neutrophils, but not with neutrophil apoptotic bodies, induced HSC activation and prolonged neutrophil survival. Mechanistic studies showed that activated HSCs produce granulocyte-macrophage colony-stimulating factor and interleukin-15 to prolong the survival of neutrophils, which may serve as a positive forward loop to promote liver damage and fibrosis.

**Conclusions:**

The current data from a mouse model of HFD plus binge ethanol feeding suggest that obesity and binge drinking synergize to promote liver fibrosis, which is partially mediated via the interaction of neutrophils and HSCs. Microarray data in this article have been uploaded to NCBI’s Gene Expression Omnibus (GEO accession number: GSE98153).

## Graphical abstract

**Figure:**
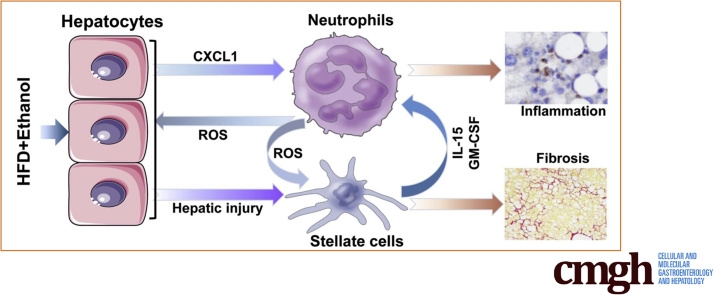

**Summary**

The present study shows that activated hepatic stellate cells produce granulocyte-macrophage colony-stimulating factor and interleukin-15 to prolong the survival of neutrophils. This may serve as a feed-forward signaling loop that promotes steatohepatitis and liver fibrosis in high-fat diet plus binge ethanol–induced steatohepatitis.

Liver disease is one of the leading global health problems, causing significant mortality worldwide. Although various risk factors have been discovered and the disease progression has been well characterized, the underlying mechanisms and therapeutic targets in various forms of liver disease are still elusive. Obesity^1^, ^2^ and alcohol drinking^3^, ^4^ are 2 well-known major risk factors for liver diseases, causing a similar spectrum of liver pathologies from simple fatty liver to more severe forms of liver injury, such as steatohepatitis, cirrhosis, and cancer. These 2 risk factors often co-exist and synergistically promote liver disease progression^5^, ^6^; however, the underlying mechanisms remain obscure. Recently, we developed a clinically relevant mouse steatohepatitis model consisting of 3-month high-fat diet (HFD) feeding plus 1 binge of ethanol (HFD+1B).^7^, ^8^, ^9^ Three-month HFD feeding alone caused obesity and severe fatty liver with a mild increase of serum alanine aminotransferase (ALT) and aspartate aminotransferase (AST) levels, but little or no hepatic neutrophil infiltration. Interestingly, acute gavage of a single dose of ethanol induced massive hepatic neutrophil infiltration and liver injury (increase of serum ALT and AST level) in 3-month HFD-fed mice,^7^, ^8^ providing a good model to study the role of neutrophils in liver injury.

Hepatic neutrophil infiltration is a critical pathologic feature in liver damage associated with both obesity and alcohol-related liver diseases.^10^, ^11^, ^12^ Neutrophils are critical components of the innate immune system. Upon infection or tissue injury, neutrophils are rapidly recruited to the infected or injured areas to clear the bacteria or remove damaged cells. However, because of the lack of selectivity, neutrophils also may cause tissue damage along with their beneficial effects on host defense.^12^, ^13^, ^14^ Hepatic neutrophil infiltration is a hallmark of alcoholic steatohepatitis in patients and also is observed in animal models of chronic plus binge ethanol feeding^15^ and HFD+1B feeding.^7^, ^8^ The results of studies using these animal models suggest that neutrophil infiltration contributes to liver injury in steatohepatitis and have identified several mechanisms underlying hepatic neutrophil infiltration in these models.^7^, ^8^, ^15^ For example, HFD+1B feeding highly up-regulated hepatic expression of chemokine (C-X-C motif) ligand 1 (CXCL1),^7^ which is one of the most selective and powerful chemokines to attract neutrophils.^16^ Inhibition of CXCL1 reduced hepatic neutrophil infiltration and ameliorated liver injury induced by HFD+1B feeding.^7^ Moreover, up-regulation of hepatic *Cxcl1* gene expression in HFD-fed mice by acute ethanol binge is partially mediated via the inhibition of hepatic peroxisome proliferator-activated receptor γ, a negative regulator for *Cxcl1* gene expression.^8^ Although HFD feeding and acute binge ethanol together are known to synergistically induce hepatic neutrophil infiltration and hepatocellular damage, whether this combined challenge also induces liver fibrosis remains unknown.

Liver fibrosis is the scarring process after liver injury, which may progress to cirrhosis and liver cancer.^17^, ^18^ Hepatic stellate cells (HSCs) are the major cell type responsible for liver fibrogenesis by producing matrix proteins during chronic liver injury.^17^, ^19^ In the quiescent state, HSCs store retinol inside their lipid droplets. During chronic liver inflammation and injury, HSCs become activated, lose lipid droplets, and differentiate into myofibroblasts, which express smooth muscle actin and various types of collagen proteins, resulting in extracellular matrix deposition and fibrosis. Over the past 4 decades, a large number of inflammatory mediators have been identified to control HSC activation.^18^ For example, activation of natural killer cells has been well documented to inhibit liver fibrosis by directly killing activated HSCs and producing interferon-γ that induces HSC apoptosis and cell-cycle arrest^20^; whereas neutrophils likely promote liver fibrosis by inducing hepatocellular damage and HSC activation via the production of reactive oxygen species (ROS).^18^ However, the exact functions of neutrophils in liver fibrogenesis in steatohepatitis have not been studied because of a lack of an animal model that recapitulates human steatohepatitis with significant neutrophil infiltration. The mouse model of steatohepatitis induced by HFD+1B feeding possesses several interesting features, including severe steatosis, a high increase of serum ALT and AST levels, and significant neutrophil infiltration in the liver.^7^, ^8^ In the current study, we performed microarray analyses using this model and found that the most robustly activated signaling pathway in the liver is related to liver fibrosis and HSC activation. Furthermore, we examined the role of neutrophils in liver fibrogenesis *in vivo* in this model, and also performed *in vitro* co-culture experiments to analyze the reciprocal interaction between neutrophils and HSCs. The data showed that apart from the already known effect of neutrophils on promoting HSC activation, activated HSCs enhanced the survival of neutrophils by producing granulocyte-macrophage colony-stimulating factor (GM-CSF) and interleukin (IL)15, thereby exacerbating liver inflammation.

## Materials and Methods

### Mice

Male C57BL/6J, intercellular adhesion molecule-1 (*Icam-1*)^-/-^, and *p47*^*phox*-/-^ mice were purchased from the Jackson Laboratories (Bar Harbor, ME) and housed in a temperature-controlled, 12-hour light/12-hour dark room. *Cxcl1*^-/-^ mice on a C57BL/6 background were kind gifts from Dr Sergio Lira (Mount Sinai, NY) and were further back-crossed into a C57BL/6J background for 5 more generations at the National Institute on Alcohol Abuse and Alcoholism animal facility, with C57BL/6J mice being used as wild-type (WT) controls. In some experiments, heterozygous breeding of *Cxcl1*^+/-^ mice was performed to obtain *Cxcl1*^-/-^ and littermate WT controls. Animal care was in accordance with the guidelines of the National Institutes of Health. The animal experiments were approved by the National Institute on Alcohol Abuse and Alcoholism Animal Care and Use Committee.

### HFD+1B or HFD-Plus-Multiple Binges of Ethanol Feeding Model

The HFD+1B feeding model in mice was described previously.^7^, ^9^ Briefly, mice were fed with an HFD diet (60% of calories as fat, catalog no. D12492; Research Diets, New Brunswick, NJ) for 3 months. For single-dose ethanol treatment, the 3-month HFD-fed mice were given 5 g/kg ethanol as a 53% ethanol solution in water by oral gavage, and were killed 9 hours later. For the maltose control group, the 3-month HFD-fed mice were given isocaloric dextrin-maltose by oral gavage, and were killed 9 hours later.

For multiple ethanol binges, the 3-month HFD-fed mice were given 5 g/kg ethanol as a 53% ethanol solution in water by oral gavage twice a week for a total of 8 times during an additional month of HFD feeding, and the mice were killed 9 hours later after the last gavage. For the maltose control group, the 3-month HFD-fed mice were given isocaloric dextrin-maltose by oral gavage twice a week for a total of 8 times during an additional month of HFD feeding, and the mice were killed 9 hours later after the last maltose gavage. The HFD plus multiple binges (HFD+mB) (5 g/kg ethanol) model has a high mortality with approximately 80% in C57BL/6N mice and approximately 30% in C57BL/6J mice, which has been described in detail by Gao et al.^9^

### Microarray Analyses of Mouse Liver Samples

Total RNAs were isolated from liver tissues of mice fed with chow diet plus 1 maltose binge (chow) (n = 4), chow diet plus 1 ethanol binge (1B) (5 g/kg, n = 4), 3-month HFD plus 1 maltose binge (HFD group) (n = 4), and 3-month HFD plus 1 ethanol binge (HFD+1B) (5 g/kg, n = 5). For each sample, 10 μg RNA samples were used for complementary DNA (cDNA) synthesis (SuperScript Double-Stranded cDNA Synthesis Kit; Thermo Fisher Scientific, Inc, Waltham, MA) and coupled with dye. A MiniElute polymerase chain reaction (PCR) purification kit (Qiagen, Germantown, MD) was used to purify dye-coupled cDNAs. The cDNA then was hybridized to an Agilent 44K mouse 60-meroligo microarray (Agilent Technologies, Santa Clara, CA). The data were analyzed with the Genespring GX software package (Agilent Technologies). Ingenuity Pathway Analysis was used to process 5 interactive Venn diagrams and gene function analyses. Full microarray data have been uploaded to NCBI’s Gene Expression Omnibus (GEO accession number: GSE98153).

### Histopathologic Analysis

Liver tissues were fixed in 10% formalin and embedded in paraffin. The 4-μm sections were further stained with H&E, Sirius Red, and Masson Trichrome (Sigma-Aldrich, St. Louis, MO). Immunohistochemical staining for myeloperoxidase (MPO) was performed with prediluted rabbit anti-MPO polyclonal antibody (cat number: PP-023 AA; Biocare Medical, LLC, Concord, CA) and a rabbit ABC staining kit (Vector Laboratories, Inc, Burlingame, CA) followed by hematoxylin staining. Immunohistochemical staining for 4-hydroxynonenal (4-HNE) was performed with mouse monoclonal anti–4-HNE antibody (Institute for the Control of Aging, Nikken SEIL Co, Fukuroi, Shizuoka, Japan) and a Mouse-on-Mouse Impress staining kit (Vector Laboratories, Inc) followed by hematoxylin staining. Immunofluorescence microscopy analysis was performed with a confocal system from Zeiss (Thornwood, NY). Terminal deoxynucleotidyl transferase–mediated deoxyuridine triphosphate nick-end labeling (TUNEL) staining was performed with an ApopTag Peroxidase In Situ Apoptosis Detection Kit (Millipore, Billerica, MA). Anti-MPO and anti-desmin (Dako, Glostrup, Demark) antibodies were used with Alexa Fluor 488– or Alexa Fluor 555–conjugated antibodies (Cell Signal Technology, Danvers, MA) for immunofluorescent staining. Hoechst 33342 (Sigma, St. Louis, MO) was used for the detection of DNA in nucleus. For quantification of Sirius Red–positive staining, at least 5 random images were taken from each mouse. The percentage of Sirius Red–positive area was determined with ImageJ software (National Institutes of Health, Bethesda, MD).

### Biochemical Assays

Serum ALT and AST levels were tested with a Catalyse Dx Chemistry Analyzer (IDEXXX Laboratories, Inc, Westbrook, ME). Hepatic collagen contents were measured by analyzing the concentration of hydroxyproline, with commercial kits from BioVision (Milpitas, CA) following the manufacturer's instructions.

### Isolation of Hepatocytes and HSCs

Primary hepatocytes and HSCs were isolated and cultured as previously described.^21^ Briefly, mice were anesthetized with sodium pentobarbital (30 mg/kg intraperitoneally) and the portal vein was cannulated. The livers were perfused with ethylene glycol-bis(β-aminoethyl ether)-*N,N,N′,N′*-tetraacetic acid solution (5.4 mmol/L KCl, 0.44 mmol/L KH_2_PO_4_, 140 mmol/L NaCl, 0.34 mmol/L Na_2_HPO_4_, 0.5 mmol/L ethylene glycol-bis(β-aminoethyl ether)-*N,N,N′,N′*-tetraacetic acid, and 25 mmol/L Tricine, pH 7.2) followed by 0.075% type I collagenase (Sigma) in Gey's Balanced Salt Solution (Sigma). After an additional digestion step with 0.009% collagenase at 37°C agitation for 30 minutes, hepatocytes were separated with 25*g* centrifugation for 5 minutes at room temperature. The precipitated hepatocytes were cultured in Dulbecco's modified Eagle medium with 10% fetal bovine serum (FBS) on collagen-coated plates from Corning (Corning, NY). The supernatant was centrifuged further at 400*g* for 10 minutes at 4°C. The cell pellet was suspended in 11.5% OptiPrep and loaded carefully with Gey's Balanced Salt Solution. After centrifugation at 1400*g* for 20 minutes at 4°C, the interface fraction was collected and further washed with Gey's Balanced Salt Solution twice. The finally collected HSCs were cultured further in RPMI-1640 medium containing 10% FBS and 10% horse serum.

### Isolation of Neutrophils

Neutrophils were obtained from the bone marrow of 8-week-old male C57B6/J mice with a neutrophil isolation kit (Miltenyi, San Diego, CA). The cell purity was more than 95%. The cells were further subject to culture.

### Co-culture of Neutrophils and HSCs

HSCs were isolated and cultured in RPMI-1640 medium with 10% FBS and 10% horse serum. One day or 5 days later, the isolated neutrophils were added (1 × 10^6^ cells per well of a 12-well-plate). The live neutrophil number was determined by using Trypan Blue staining 1–4 days later. The neutrophils were analyzed further with Wright-Giemsa staining (Sigma-Aldrich) and TUNEL staining (Millipore, Cleveland, OH). The HSC conditioned medium was prepared 24 hours after the refreshment of the medium of the 5-day cultured HSCs. When indicated, neutralizing antibodies against GM-CSF (cat number: MAB415-100; R&D, Minneapolis, MN) and IL-15 (cat number: AF447-SP; R&D) were applied to the co-culture system.

### Engulfment Experiment

Bone marrow neutrophils and HSCs were isolated from the same mice. Neutrophils were fluorescently labeled with 5-Carboxytetramethylrhodamine Succinimidyl Ester (Molecular Probes, Inc, Eugene, OR) 10 μmol/L for 30 minutes at 37°C before induction of apoptosis by UV light (0–100 mJ/cm^2^ for 142 s). After 24 hours, the floating apoptotic bodies were collected by centrifugation at 2000*g* for 4 minutes and resuspended in Dulbecco's modified Eagle medium. HSCs were synchronized at 4°C for 10 minutes, then incubated for 24 hours at 37°C with fluorescent apoptotic bodies. Approximately 900 apoptotic bodies/mL were used for these experiments. Engulfment of fluorescent apoptotic bodies was studied by fluorescent microscopy; cells in 5 different fields were counted, and the ratio of engulfed apoptotic bodies was calculated.

### Enzyme-Linked Immunosorbent Assay Analysis of GM-CSF

The concentration of GM-CSF in the culture supernatant of HSCs was measured with a GM-CSF enzyme-linked immunosorbent assay kit (R&D) according to the manufacturer's instructions.

### Real-Time Quantitative PCR

Liver tissue or cell culture samples underwent total RNA extraction with TRIzol Reagent (Invitrogen, Carlsbad, CA) according to the manufacturer’s instructions, followed by cDNA synthesis using the High-Capacity cDNA Reverse Transcription Kit (Applied Systems, Foster City, CA). Real-time PCR analysis of gene expression was performed with the ABI PRISM 7500 Real-time PCR system (Applied Biosystems). The reaction mixture contained 3 μL cDNA (equal to 50 ng RNA), 0.5 μmol/L forward and reverse primers, and 10 μmol/L SYBR Green Master Mix (Abm, Ontario, Milton, Canada) adjusted to a total of 20 μL. Amplification was performed at 50°C for 2 minutes, 95°C for 10 minutes, followed by 40 cycles at 95°C for 15 seconds, and 60°C for 1 minute followed by melting curve profiling to confirm specificity. Target gene expression levels were calculated with normalization to 18S followed by a comparative cycle threshold Ct method (2-ΔΔCt). The primer sequences are shown in Table 1.

**Table 1:** Real-Time Quantitative PCR Primer Sequence Used in the Present Study

Gene name	Forward	Reverse
*Acta2* (encodes α-SMA)	TCCTGACGCTGAAGTATCCGATA	GGTGCCAGATCTTTTCCATGTC
*collagen 1a1*	TAGGCCATTGTGTATGCAGC	ACATGTTCAGCTTTGTGGACC
*collagen 1a2*	GGTGAGCCTGGTCAAACGG	ACTGTGTCCTTTCACGCCTTT
*collagen 3a1*	TAGGACTGACCAAGGTGGCT	GGAACCTGGTTTCTTCTCACC
*collagen 4a1*	CACATTTTCCACAGCCAGAG	GTCTGGCTTCTGCTGCTCTT
*collagen 5a2*	CATGGAGAAGGTTTCCAAATG	AAAGCCCAGGAACAAGAGAA
*collagen 12a1*	TGAGGTCTGGGTAAAGGCAA	GTATGAGGTCACCGTCCAGG
*Ly6g*	TGCGTTGCTCTGGAGATAGA	CAGAGTAGTGGGGCAGATG
*Csf2* (encodes GM-CSF)	CTGCTCTTCTCCACGCTACTG	GAGACTCGCCGGTGTATCC
*Csf3* (encodes G-CSF)	CTGATCTTCTTGCTACTCCCCA	GGTGTAGTTCAAGTGAGGCAG
*IL3*	GGGATACCCACCGTTTAACCA	AGGTTTACTCTCCGAAAGCTCTT
*IL15*	ACATCCATCTCGTGCTACTTGT	GCCTCTGTTTTAGGGAGACCT
*Egf*	AGCATCTCTCGGATTGACCCA	CCTGTCCCGTTAAGGAAAACTCT
*Hgf*	ATGTGGGGGACCAAACTTCTG	GGATGGCGACATGAAGCAG
*IL17a*	TTTAACTCCCTTGGCGCAAAA	CTTTCCCTCCGCATTGACAC
*IL17f*	TGCTACTGTTGATGTTGGGAC	AATGCCCTGGTTTTGGTTGAA
*Ly6g*	TGCGTTGCTCTGGAGATAGA	CAGAGTAGTGGGGCAGATGG
18s	GTAACCCGTTGAACCCCATT	CCATCCAATCGGTAGTAGCG

α-SMA, α-smooth muscle actin; Egf, epidermal growth factor; G-CSF, granulocyte colony-stimulating factor; Hgf, hepatocyte growth factor.

### Statistical Analysis

Data are shown as means ± SEM. Data from 2 groups were compared with an unpaired *t* test. Data from multiple groups were compared with 1-way analysis of variance followed by the Tukey post hoc test. *P* < .05 was considered a significant difference.

### Data

All authors had access to the study data and reviewed and approved the final manuscript.

## Results

### Microarray Analyses Show Strong Activation of Fibrosis and Neutrophilic Pathways in the Liver From Mice With HFD+1B Ethanol Challenge

**Figure 1:**
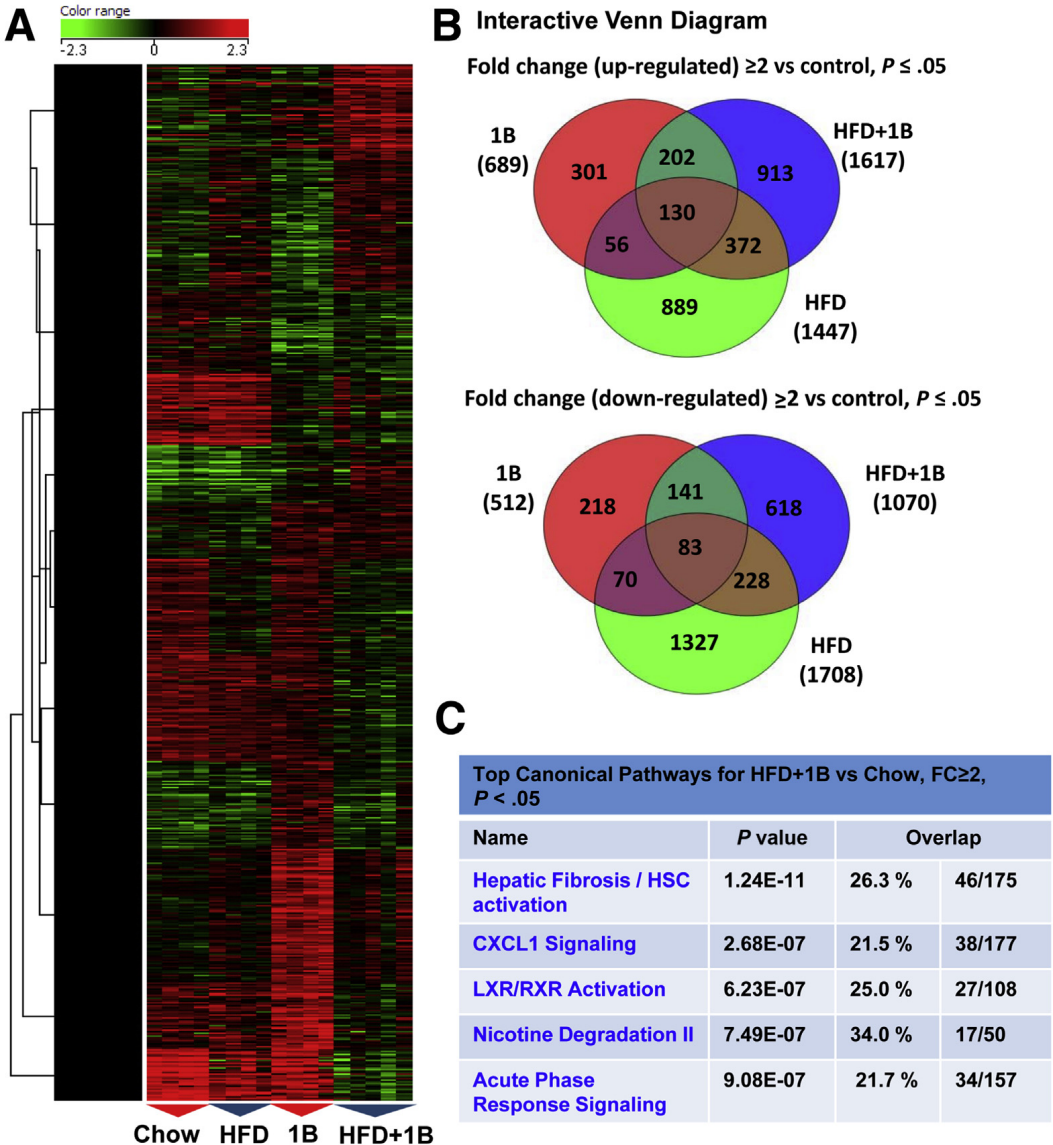
**Acute ethanol binge and 3-month HFD feeding synergistically activate hepatic fibrosis and neutrophilic pathways.** Microarray analysis was performed in the liver tissues from mice subjected to chow diet plus maltose gavage (chow), acute ethanol binge (5 g/kg, 1B), high-fat diet plus maltose gavage (HFD), or HFD-plus-ethanol binge (HFD+1B). (*A*) Heat map and (*B*) interactive Venn diagram analyses for genes showing ≥2 fold up-regulation or down-regulation compared with control in microarray data were shown. (*C*) Top 5 most highly increased pathways in HFD+1B vs chow control mice were listed. N = 4–5 in each group. LXR, liver X receptor; RXR, retinoid X receptor.

We previously showed that 3-month HFD+1B induced significant liver injury, as shown by marked increases of serum ALT and AST levels.^7^, ^8^ To further understand the underlying molecular mechanisms, we analyzed hepatic gene expression by microarray in this model. Heat map (Figure 1*A*) and interactive Venn diagram (Figure 1*B*) analyses showed great differences in gene expression profile in various groups. Because HFD+1B challenge synergistically induced severe liver injury and liver inflammation whereas HFD or 1B alone only induced very mild liver injury, we further performed the pathway analyses in the differential expression genes of HFD+1B vs chow-fed mice. The results showed that the most strongly increased signal pathway was related to hepatic fibrosis and HSC activation (Figure 1*C*), suggesting a robust hepatic fibrogenic process in this model.

### HFD+1B/mB Ethanol Challenge Induces Liver Fibrosis in Mice

**Figure 2:**
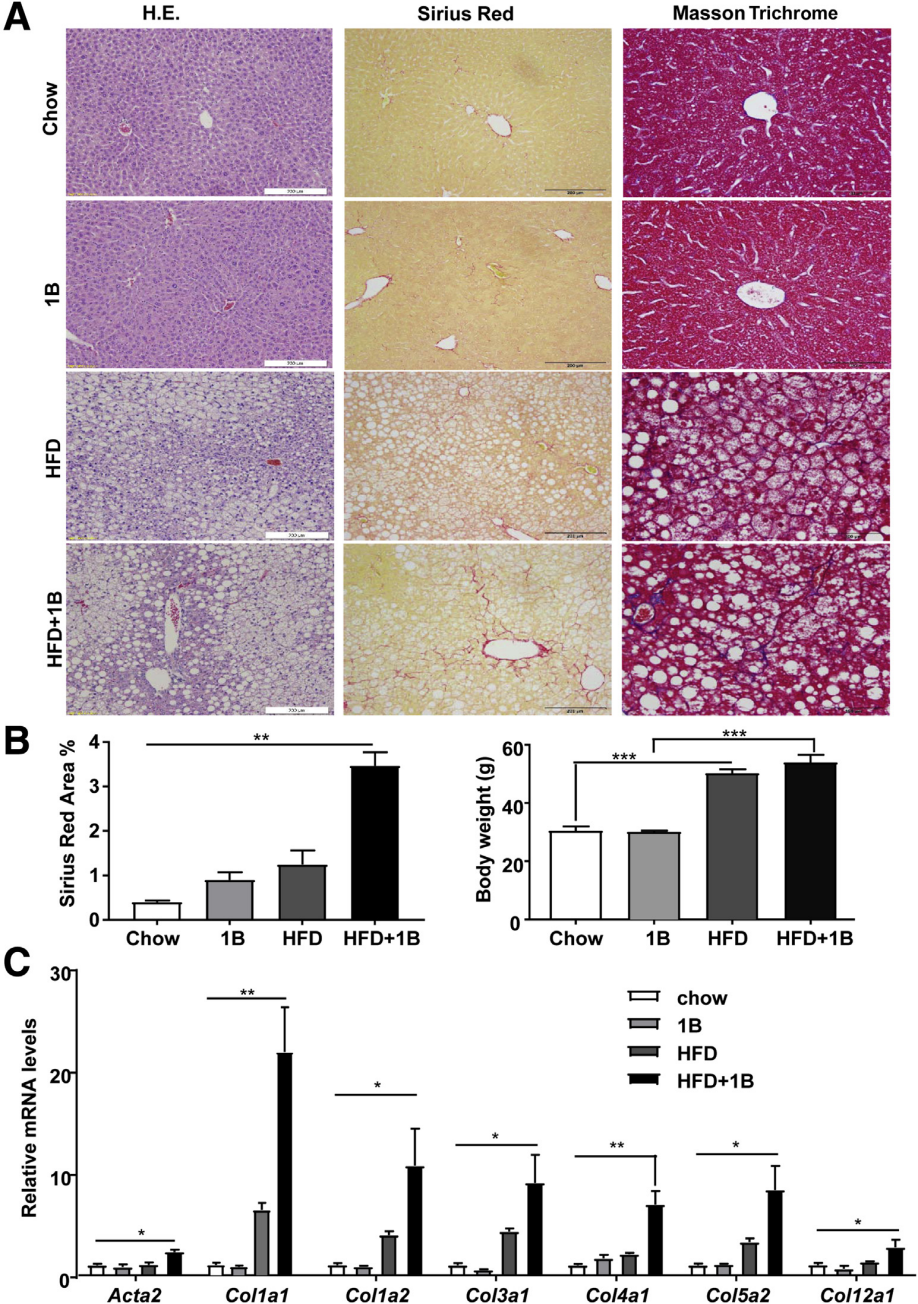
**HFD-plus-one binge ethanol challenge induces liver fibrosis.** Mice were subject to chow diet plus maltose gavage (chow), acute ethanol binge (5 g/kg, 1B), high-fat diet plus maltose gavage (HFD), or HFD-plus-ethanol binge (HFD+1B). Mice were euthanized 9 hours after gavage, and then liver samples were collected and analyzed. (*A*) Representative images of H&E staining, Sirius Red and Masson Trichrome staining of liver tissues. (*B*) Sirius Red–positive area was quantified, and body weight was measured. (*C*) Liver tissues were collected for quantitative RT-PCR analyses of fibrogenic genes. N = 6–7 in each group. *P < .05, **P < .01, ***P < .001.

Liver fibrosis was further confirmed by Sirius Red, Masson Trichrome staining, and reverse-transcription PCR (RT-PCR) analyses of fibrogenic genes. As illustrated in Figure 2*A* and *B*, a more pronounced chicken wire fibrosis was shown by Sirius Red or Masson Trichrome staining in the HFD+1B compared with chow or HFD groups, whereas body weight gain was comparable between these 2 groups. Real-time PCR analyses in Figure 2*C* showed that the HFD+1B group had higher expression levels of fibrosis-related genes, including multiple collagen genes and α-smooth muscle actin (*Actn2*) compared with chow or HFD group. Surprisingly, hepatic collagen levels were slightly increased in the HFD+1B group compared with the HFD group, but did not reach statistical difference (data not shown), which is probably because high basal levels of collagen in the liver structure overwhelmed the mild increase of collagen observed in this mild liver fibrosis model of HFD+1B feeding.

**Figure 3:**
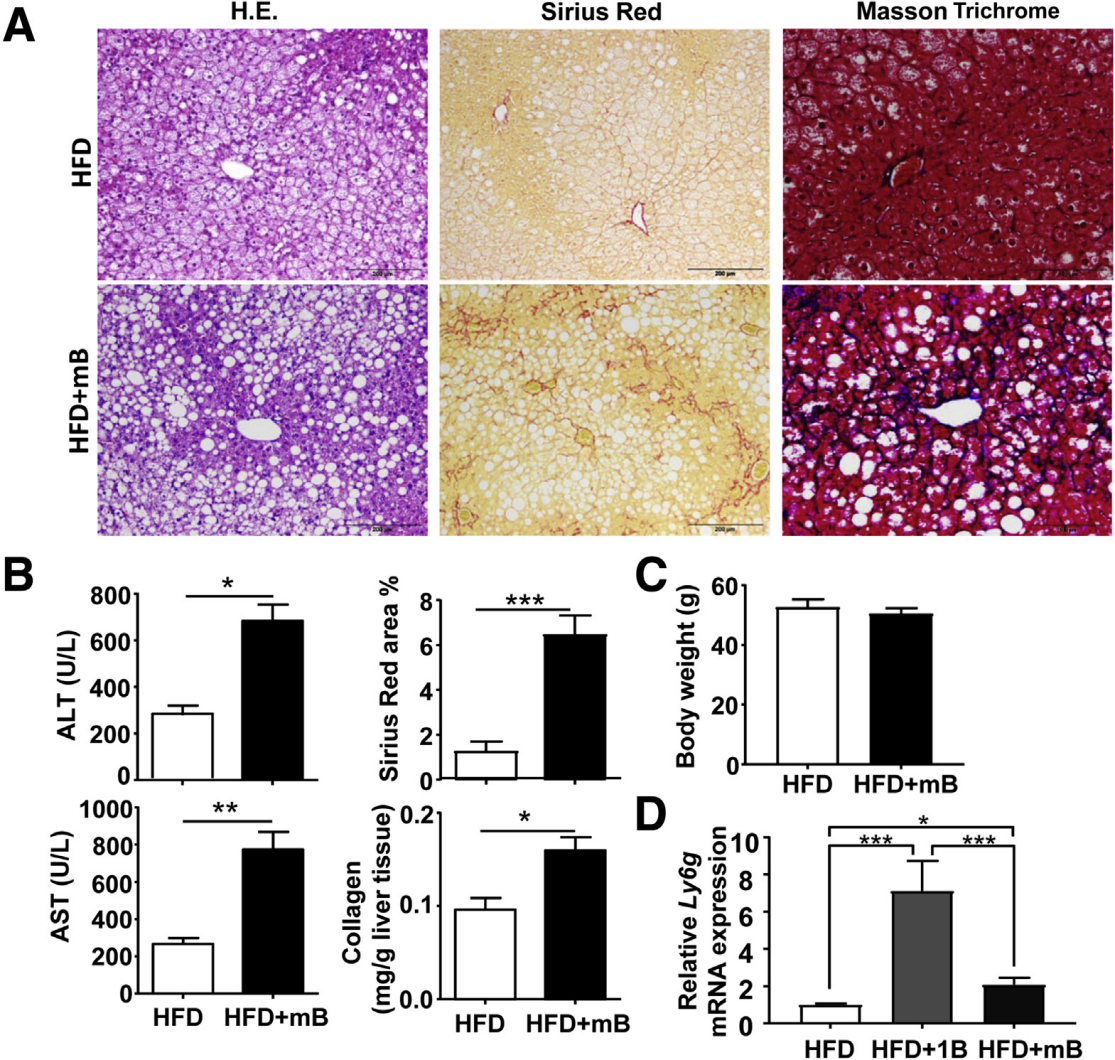
**HFD-plus-multiple binges of ethanol challenge induces liver fibrosis.** (*A–C*) Male C57BL/6J mice were fed an HFD for 3 months, followed by 8 gavages of 5 g/kg ethanol (twice a week) (HFD+mB group), or isocaloric dextrin-maltose (HFD group), while continuing on HFD for an additional month. (*A*) Representative images are shown. (*B* and *C*) Serum ALT and AST levels, liver tissue collagen content, percentage of Sirius Red–positive area, as well as body weight were examined and are shown. (*D*) Liver tissues from different groups were subject to quantitative RT-PCR analyses of *Ly6g* mRNA. N = 6–7 in each group. *P < .05, **P < .01, ***P < .001.

Because liver fibrosis is typically a chronic pathologic process in response to repeated or sustained injury, we performed HFD+mB ethanol gavages. As shown in Figure 3*A*, HFD+mB ethanol binges caused significant liver fibrosis, as shown by Sirius Red and Masson Trichrome staining. Moreover, serum ALT and AST levels and hepatic collagen contents were higher in the HFD+mB group than in the HFD-plus-multiple maltose binges group (Figure 3*B*); whereas body weight was comparable between these 2 groups (Figure 3*C*). HFD+1B and HFD+mB mice had approximately 3-fold and 6-fold increases in Sirius Red–positive area, respectively, compared with their HFD+maltose groups (Figures 2*B* and 3*B*), suggesting that HFD+mB mice had higher levels of liver fibrosis than HFD+1B mice. Interestingly, quantitative RT-PCR analyses of the neutrophil marker *Ly6g* messenger RNA (mRNA) showed that hepatic neutrophils were increased much higher in HFD+1B compared with those in HFD+mB mice (Figure 3*D*). These results indicate that acute binge ethanol highly increased hepatic neutrophil infiltration in HFD-fed mice, while chronic binges of ethanol caused less hepatic neutrophil infiltration in these mice, which may be because the multiple binge model has passed the peak neutrophil infiltration phase.

### Evidence for the Involvement of Neutrophils in the Pathogenesis of Liver Fibrosis Induced by HFD+1B Ethanol Feeding

Our previous work documented that neutrophils play an important role in inducing liver damage in the HFD+1B ethanol feeding model.^7^ To test whether neutrophils also contribute to the liver fibrosis in this model, we used 2 approaches to attenuate neutrophil infiltration via the inhibition of CXCL1 or ICAM-1, 2 important mediators for neutrophil infiltration.^16^

**Figure 4:**
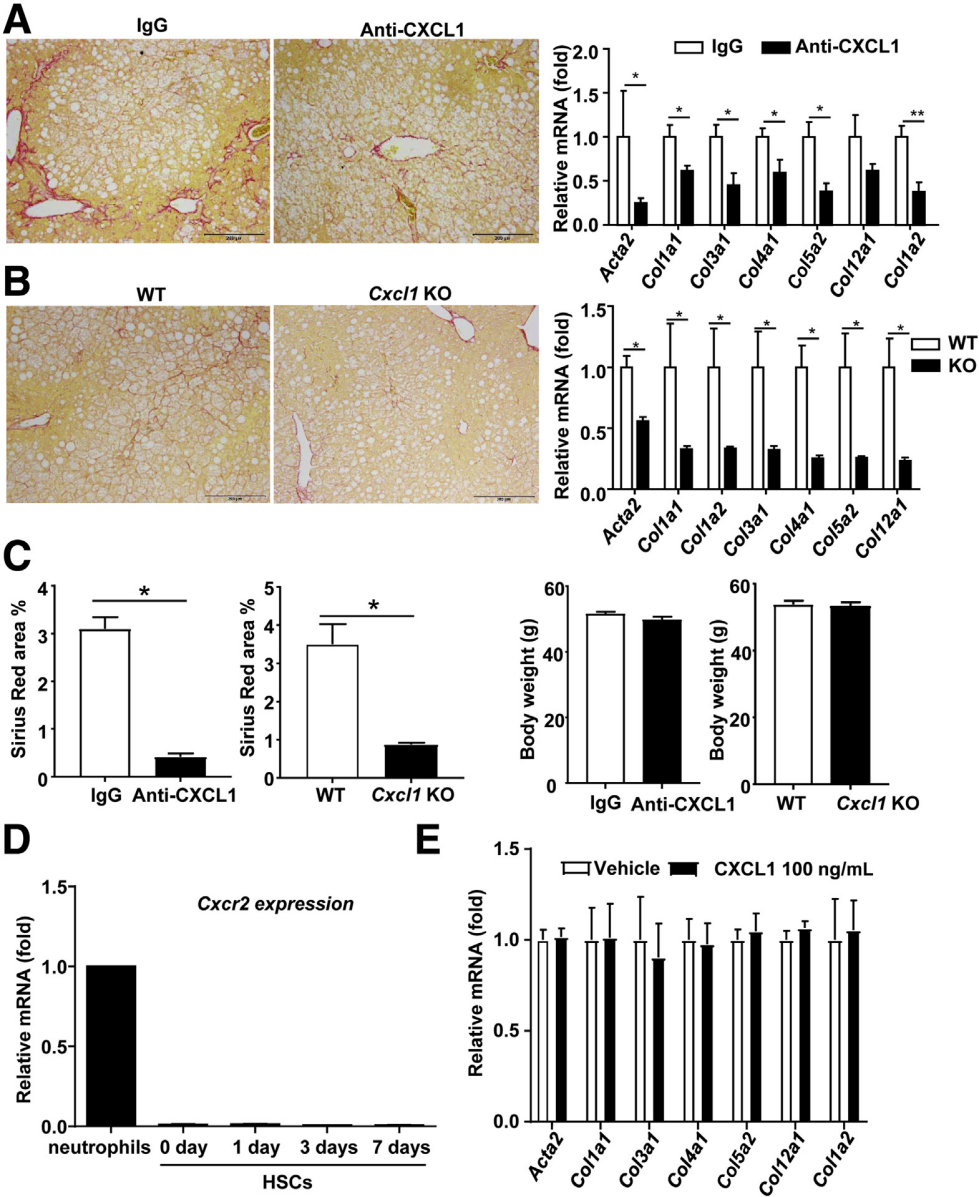
**CXCL1 plays an important role in promoting liver fibrosis induced by HFD-plus-binge ethanol feeding model.** (*A*) Male C57BL/6J mice were fed an HFD for 3 months and then injected intraperitoneally with control IgG or CXCL1 neutralizing antibody. Fifteen minutes later, mice were gavaged with 5 g/kg ethanol. Nine hours later the tissue samples were analyzed with Sirius Red staining and quantitative RT-PCR for fibrosis. (*B*) WT C57B/6J mice and *Cxcl1* KO mice were subject to HFD+1B ethanol challenge, and mice were euthanized 9 hours after gavage. The liver samples were analyzed with Sirius Red staining and quantitative RT-PCR. *Acta2* gene: encodes α-smooth muscle actin. Images represent 1 of 5 fields in each group. (*C*) Percentage of Sirius Red–positive area and body weight in panels *A* and *B* were statistically analyzed. (*D*) HSCs were isolated from C57BL/6J mice and then cultured, and subject to quantitative RT-PCR analysis of *Cxcr2* mRNA. Neutrophils were used as a positive control analysis of *Cxcr2* mRNA. (*E*) Cultured HSCs were incubated with 100 ng/mL CXCL1 or vehicle. Five days later, HSCs were collected and subject to quantitative RT-PCR analyses of fibrosis-related genes. (*A*–*C*) N = 5–6. (*D*) Data were from 2 independent experiments. (*E*) N = 3. *P < .05, **P < .01.

As shown in Figure 4*A–C*, blockade of CXCL1 with a neutralizing antibody or genetic disruption of the *Cxcl1* gene markedly attenuated liver fibrosis in the HFD+1B ethanol-fed mice relative to IgG-treated or WT controls, as shown by the reduced Sirius Red–positive area and fibrosis-related gene expression. In addition, body weight gain was comparable between IgG- and anti-CXCL1 antibody-treated mice, and between WT and *Cxcl1* knockout (KO) mice after HFD+1B feeding.

We next wanted to know whether CXCL1 can directly affect HSC activation by analyzing the effect of CXCL1 on isolated HSCs. The CXCL1 receptor *Cxcr2* mRNA was expressed at very low levels in freshly isolated and cultured HSCs, whereas its expression was much higher in neutrophils (Figure 4*D*). Moreover, *in vitro* treatment with CXCL1 had no effect on the expression of collagens in cultured HSCs (Figure 4*E*), suggesting that CXCL1 does not directly induce HSC activation.

**Figure 5:**
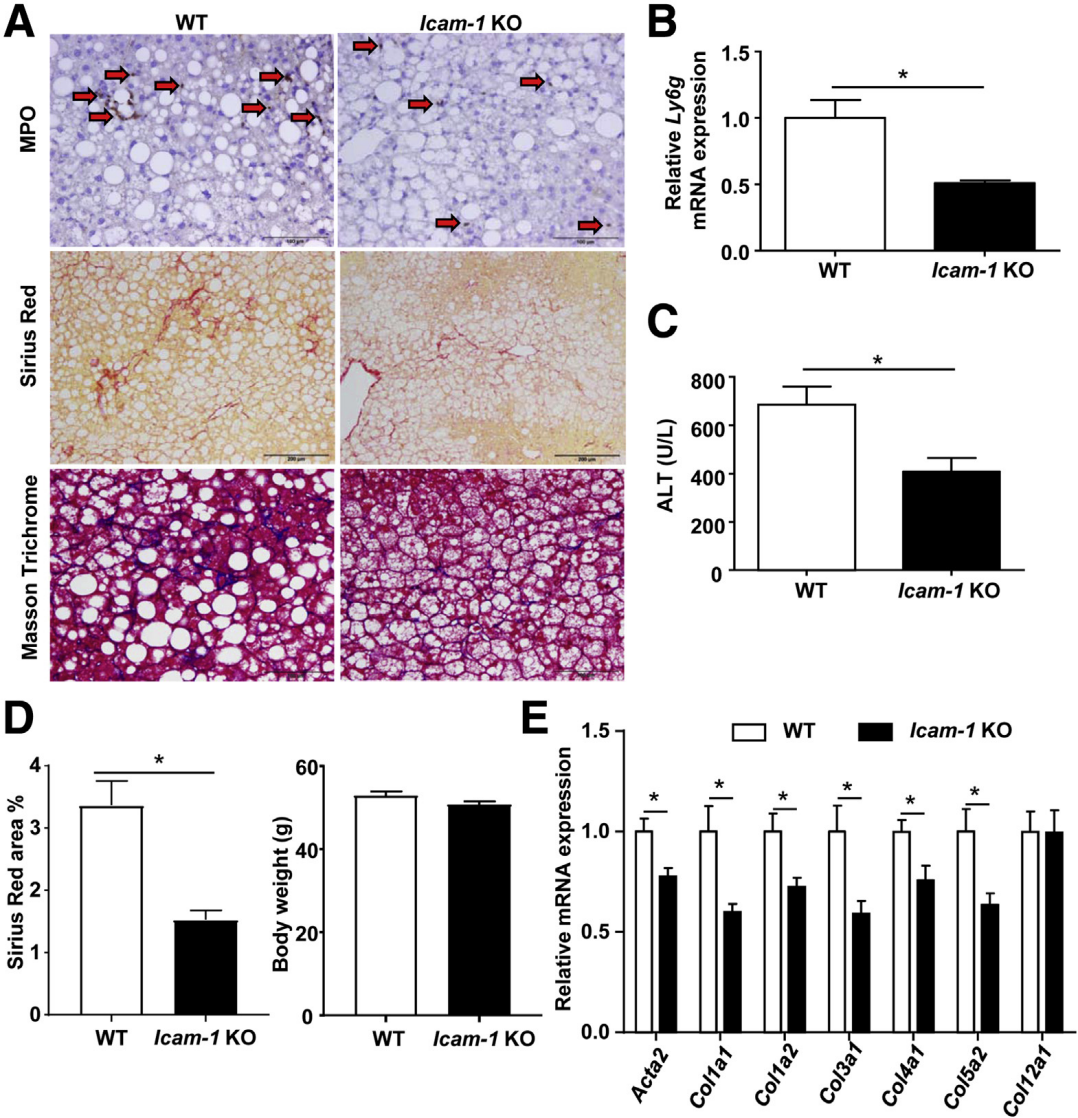
***Icam-1*–deficient mice have reduced hepatic neutrophil infiltration, liver injury, and fibrosis induced by HFD+1B ethanol feeding.** WT and *Icam-1* KO mice were subject to HFD+1B ethanol challenge, and mice were euthanized 9 hours after gavage. (*A*) Liver sections were stained with MPO, Sirius Red, and Masson Trichrome. Images represent 1 of 5 fields in each group. *Arrows* indicate MPO^+^ cells. (*B*) Quantitative RT-PCR analyses of neutrophil marker gene *Ly6g*. (*C*) Serum ALT levels were analyzed. (*D*) Percentage of Sirius Red–positive area and body weight were analyzed. (*E*) Quantitative RT-PCR analyses of fibrosis-related genes. (*B–E*) N = 5–7 in each group. *P < .05.

As expected, the *Icam-1* KO mice had reduced hepatic neutrophil infiltration compared with WT mice, as shown by the lower number of MPO^+^ neutrophils (Figure 5*A*) and lower levels of *Ly6g* mRNA (Figure 5*B*). Serum ALT levels were lower in the *Icam-1* KO mice than those in WT mice (Figure 5*C*). In addition, Sirius Red and Masson Trichrome staining showed that *Icam-1* KO mice had lower levels of liver fibrosis than WT mice (Figure 5*A*), whereas body weight was comparable between these 2 groups (Figure 5*D*). Hepatic expression of several fibrogenic genes was also lower in *Icam-1* KO mice than that in WT mice (Figure 5*E*).

### ROS Production From Neutrophils Contributes to Liver Fibrosis

**Figure 6:**
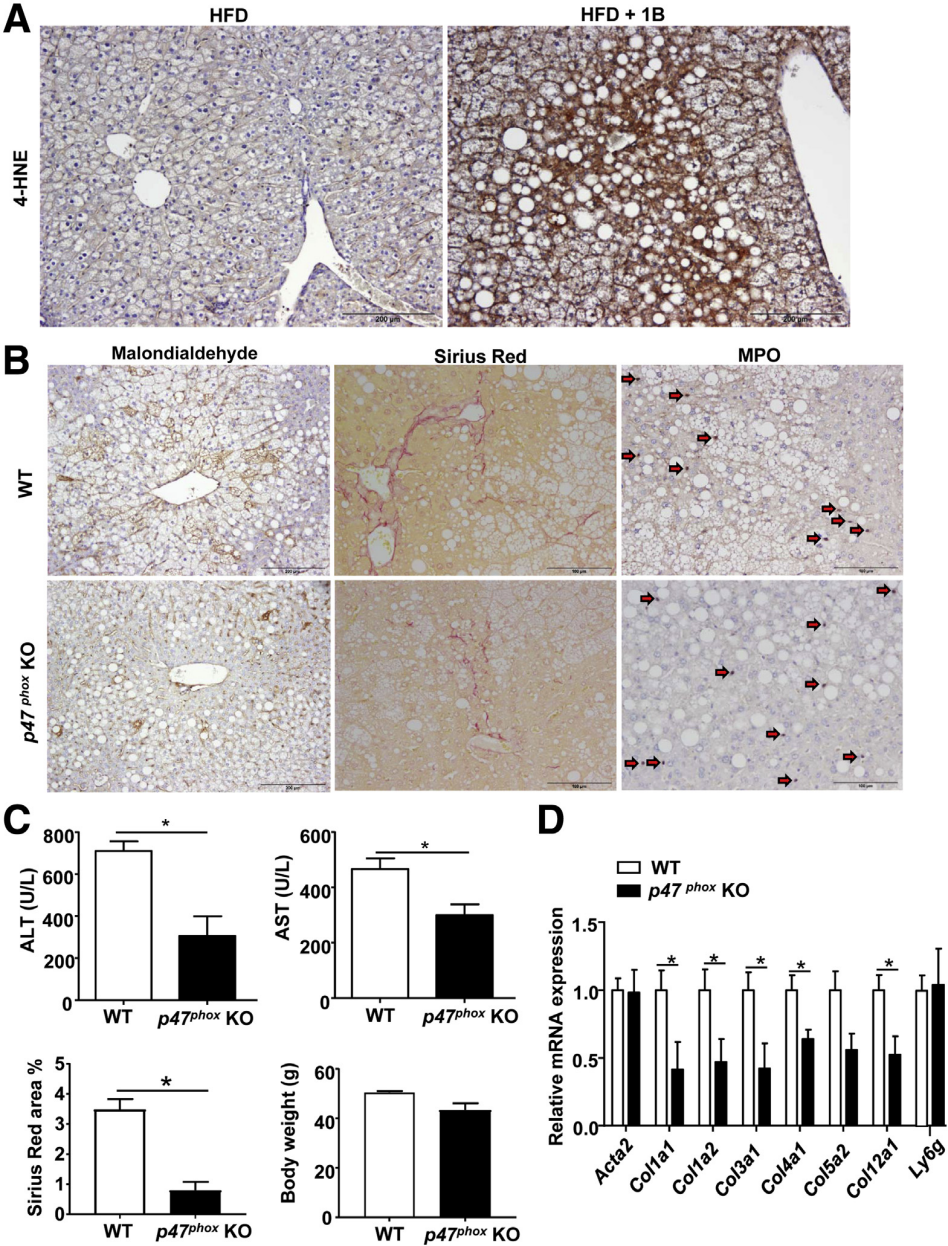
***P47***^***phox***^**KO mice have reduced liver damage and fibrosis in the HFD+1B ethanol model.** (*A*) C57BL/6J mice were subject to HFD+1B ethanol or HFD-plus-maltose (HFD group) challenge. Liver tissues were collected and subject to 4-HNE staining. (*B–D*) WT and *p47*^*phox*^ KO mice were subject to HFD+1B ethanol challenge. Malondialdehyde Sirius Red, and MPO staining were performed on the liver tissue sections. (*B*) Representative images are shown, *arrows* indicate MPO^+^ cells). (*C*) Serum ALT and AST levels, percentage of Sirius Red–positive area, as well as body weight were measured. (*D*) Quantitative RT-PCR analyses of liver fibrosis-related genes and *Ly6g* mRNA. N = 4–7 in each group, *P < .05.

Our data suggest that neutrophils not only induce hepatocellular damage but also promote liver fibrosis in the HFD+1B ethanol feeding model. It is known that neutrophils induce hepatocellular damage by killing hepatocytes via the production of ROS.^22^ Here, we showed that HFD+1B ethanol-fed mice, which are known to have massive hepatic neutrophil infiltration,^7^ showed strong hepatic 4-HNE (a marker of lipid peroxidation/ROS) staining, whereas 4-HNE staining was absent in the liver from HFD-plus-maltose binged mice (Figure 6*A*). Thus, HFD+1B ethanol challenge promotes ROS production in the liver. To better define the role of ROS in the liver damage and fibrosis in this model, we used p47^phox^ KO mice. p47^phox^, a critical component of the phagocytic reduced nicotinamide adenine dinucleotide phosphate oxidase, is expressed predominately in neutrophils, where it plays a key role in generating oxidative bursts.^23^ As shown in Figure 6*B–D*, p47^phox^ KO mice had lower levels of hepatic malondialdehyde (ROS marker), Sirius Red staining, and serum ALT and AST compared with WT mice after HFD-plus-binge ethanol challenge, whereas MPO staining and quantitative RT-PCR analysis of neutrophil marker *Ly6g* showed that the number of hepatic neutrophils was comparable between WT and p47^phox^ KO mice. Finally, body weight gain was comparable between WT and p47^phox^ KO mice after HFD+1B challenge (Figure 6*C*).

### Engulfment of Neutrophil Apoptotic Bodies Does Not Activate HSCs

**Figure 7:**
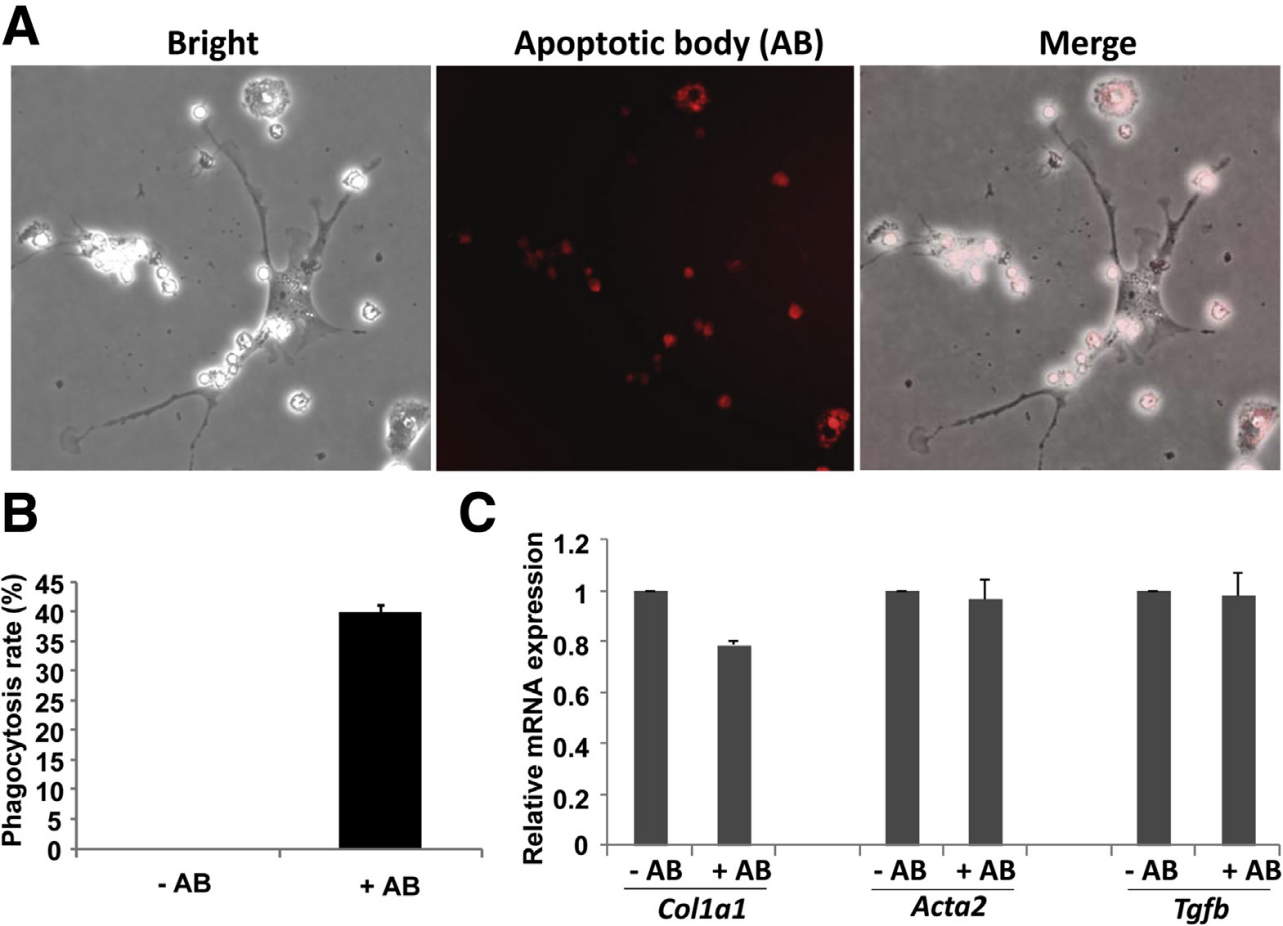
**Engulfment of neutrophil apoptotic body (AB) does not activate HSCs.** Neutrophils from the bone marrow and HSCs from the liver were isolated. The neutrophils were labeled with 5-Carboxytetramethylrhodamine Succinimidyl Ester and exposed to UV to generate apoptotic bodies. These apoptotic bodies were incubated with HSCs for 24 hours. (*A* and *B*) Engulfment was evaluated under a microscope. (*A*) Representative images are shown. (*B*) The phagocytosis/engulfment rate was calculated as the percentage of cells with intracellular 5-Carboxytetramethylrhodamine Succinimidyl Ester-labeled apoptotic bodies. (*C*) HSCs from panel *B* then were collected for quantitative RT-PCR assay to evaluate the expression of Col1a1, α-smooth muscle actin, and transforming growth factor β (Tgfb).

Previous studies have documented that HSCs can engulf the apoptotic bodies of hepatocytes and subsequently become activated.^24^, ^25^ Neutrophils are short-lived cells and become apoptotic after activation. Indeed, apoptotic neutrophils frequently were observed in the livers from mice with HFD+1B or HFD+mB mice (data not shown). Thus, we hypothesized that HSCs become activated by engulfing neutrophil apoptotic bodies. To test this hypothesis, HSCs were incubated with neutrophil apoptotic bodies. Our data showed that HSCs significantly engulfed neutrophil apoptotic bodies (Figure 7*A* and *B*), but HSC activation was not enhanced because the expression of fibrosis-related genes were not increased after the engulfment of neutrophil apoptotic bodies (Figure 7*C*).

### Activated HSCs Reduce the Spontaneous Apoptosis of Neutrophils Through the Production of GM-CSF and IL-15

**Figure 8:**
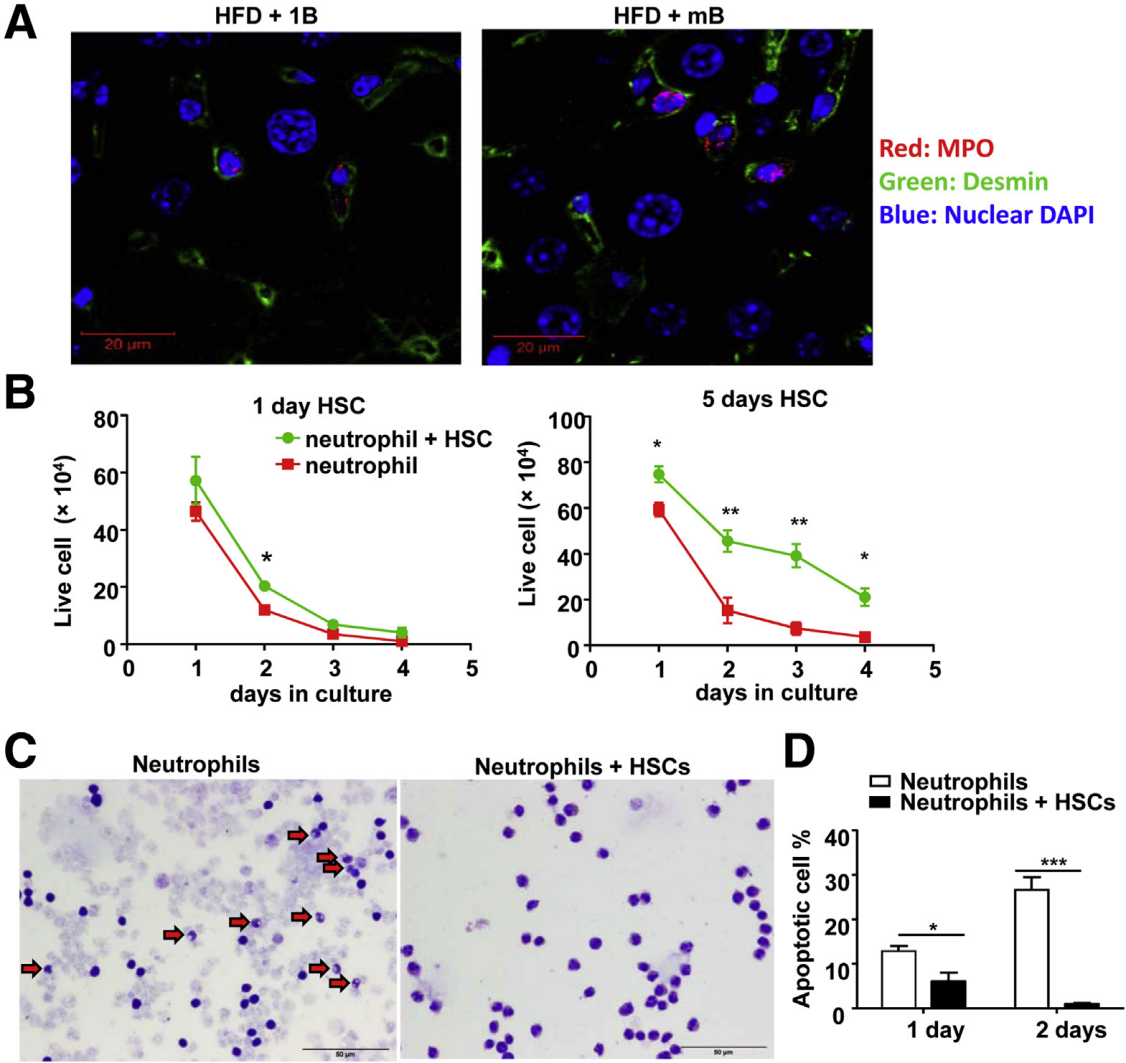
**Co-culture with HSCs reduces apoptosis and promotes survival of neutrophils.** (*A*) Confocal immunofluorescence staining of MPO and desmin (a marker for HSCs) in the liver sections of HFD+1B or HFD+mB ethanol-fed mice. Only a few number of MPO^+^ cells were observed in this figure owing to extremely high magnification from confocal microscope analysis. (*B*) Mouse bone marrow neutrophils were cultured alone or with 1-day cultured HSCs (*left panel*) or 5-day cultured HSCs (*right panel*). The number of live neutrophils was counted and statistically analyzed. (*C*) Wright–Giemsa staining of the neutrophils cultured for 2 days with or without HSCs. *Arrows* indicate apoptotic neutrophils determined according to the morphology. (*D*) Statistical analysis of TUNEL^+^ neutrophils after culture for 1 or 2 days with or without HSCs. (*A* and *C*) Images represent 1 of at least 5 randomly selected fields. n = 3–4 in each group, *P < .05, **P < .01, ***P < .001. DAPI, 4′,6-diamidino-2-phenylindole.

To further test whether HSCs and neutrophils interact with each other, we performed immunostaining to determine the co-localization of neutrophils and HSCs. As shown in Figure 8*A*, co-localization of neutrophils (red staining) and HSCs (green staining) was clearly observed in the livers of HFD+ single or multiple ethanol binges.

Neutrophils are known to activate HSCs via the production of ROS.^26^, ^27^ Here, we found that neutrophils underwent spontaneous apoptosis when they were cultured *in vitro*. Interestingly, co-culture with HSCs, especially day 5 HSCs, markedly increased the survival of neutrophils (Figure 8*B*). Neutrophils cultured by themselves showed a lot of cell debris and apoptotic features such as a shrinking and dissolving nucleus, whereas neutrophils co-cultured with activated day 5 HSCs were generally healthy as visualized by Wright–Giemsa staining (Figure 8*C*). Moreover, TUNEL staining showed that the percentage of TUNEL^+^ apoptotic neutrophils was lower in cells co-cultured with activated HSCs for 1 or 2 days compared with neutrophils cultured by themselves (Figure 8*D*).

**Figure 9:**
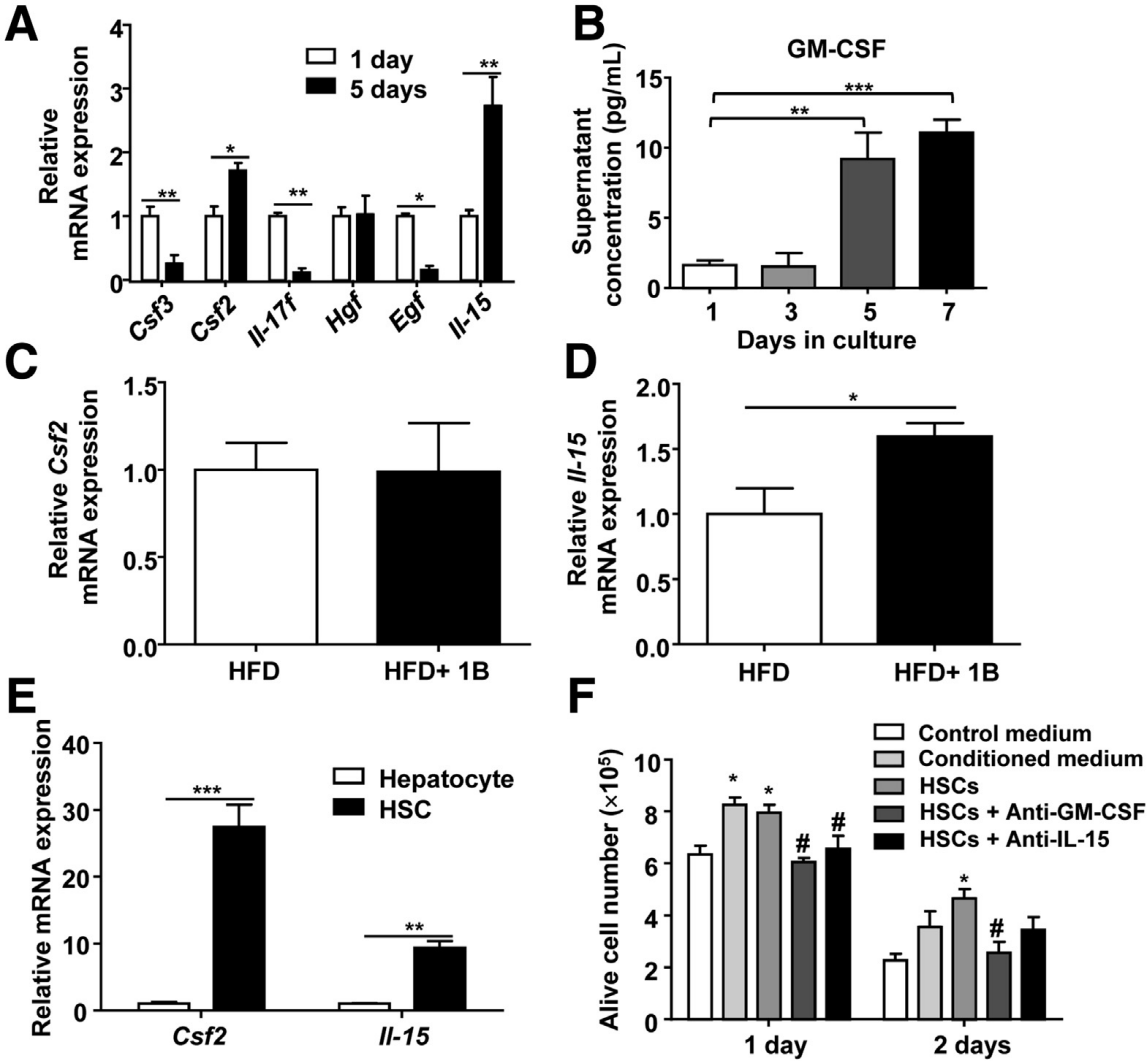
**HSCs delay the spontaneous apoptosis of cultured neutrophils via the secretion of GM-CSF and IL-15.** (*A*) Quantitative RT-PCR analyses of mRNA expression levels of cytokines and growth factors in 1-day or 5-day cultured HSCs. N = 3 in each group. (*B*) Concentrations of GM-CSF from cultured HSCs were analyzed with an enzyme-linked immunosorbent assay. Values were obtained from 4 independent experiments. (*C* and *D*) Quantitative RT-PCR analyses of *Csf2* and *Il15* mRNA expression levels from HFD+1B vs HFD groups. N = 4–6 in each group. (*E*) *Csf2* and *Il15* expression in primary hepatocytes and HSCs after 1-day culture. N = 3–7 in each group. (*F*) Neutrophils were cultured with 5-day cultured HSCs or HSC-conditioned medium as well as neutralizing antibodies against GM-CSF or IL-15 as indicated. The number of live cells was counted and statically analyzed. N = 4–8 in each group. *P < .05, **P < .01, ***P < .001 vs control medium group, ^#^*P* < .05 vs neutrophils + HSCs group. Egf, epidermal growth factor; Hgf, hepatocyte growth factor.

To analyze the mechanisms by which activated HSCs delayed the spontaneous apoptosis of neutrophils, we examined the expression of several cytokines or growth factors that are known to improve the survival of neutrophils. As shown in Figure 9*A*, expression of colony stimulating factor (*Csf*)*3*, which encodes G-CSF, *Il17f*, and *Egf* was lower, whereas expression of *Csf2*, which encodes GM-CSF, and *Il15* was significantly higher in activated HSCs compared with quiescent HSCs. Enzyme-linked immunosorbent assay analyses showed that cultured HSCs produced GM-CSF proteins (Figure 9*B*). Expression of *Hgf* mRNA remained unchanged (Figure 9*A*) and expression of *Il3* and *Il17a* was undetectable (data not shown). Moreover, hepatic expression of *csf2* was not up-regulated, whereas *Il15* expression was increased in mice exposed to HFD+1B feeding (Figure 9*C* and *D*). Finally, compared with hepatocytes, HSCs expressed much higher levels of *Csf2* and *Il15* (Figure 9*E*).

To further determine whether HSC-derived GM-CSF and IL-15 protected against neutrophil apoptosis, neutralizing antibodies against these cytokines were used. As shown in Figure 9*F*, treatment with GM-CSF or IL-15 neutralizing antibodies reduced neutrophil survival in co-culture with HSCs.

## Discussion

Obesity and alcohol abuse are 2 major predisposing factors for liver diseases. Our previous study established a clinically relevant HFD-plus-binge ethanol feeding model to explore the synergistic effect of these 2 risk factors on liver injury,^7^, ^8^, ^9^ and showed a critical role for neutrophils in the hepatocellular damage in this model.^7^ In the present study, we performed microarray analyses and identified that the most up-regulated pathway after HFD-plus-binge ethanol challenge was related to liver fibrosis and HSC activation. Further analyses showed that neutrophils and HSCs reciprocally regulate each other to promote liver fibrogenesis.

It is well established that feeding mice with an HFD diet alone causes massive steatosis but little liver fibrosis. One of the noteworthy findings in the present study was that adding a single or multiple binges of ethanol to chronic HFD feeding resulted in significant fibrosis, as shown by Sirius Red staining, RT-PCR analyses of fibrogenesis-related genes, as well as microarray analyses. We also provide several lines of evidence to indicate that neutrophils contribute to liver fibrosis in this steatohepatitis model. First, blockade of CXCL1, a critical chemokine to attract neutrophils, via the disruption of the *Cxcl1* gene or treatment with a neutralizing anti-CXCL1 antibody, reduced hepatic neutrophil infiltration and liver fibrosis. Second, disruption of the *Icam-1* gene that encodes ICAM-1 protein, a key adhesion molecule for neutrophil recruitment,^16^ also attenuated neutrophil infiltration and liver fibrosis in this model. The possible contribution of neutrophils to liver fibrosis was postulated based on results using an *in vitro* culture system in which neutrophils activate the HSCs to express collagen genes through the production of ROS.^26^ In the current study, we show that disruption of the *p47*^*phox*^ gene, which encodes an important ROS-producing enzyme in neutrophils,^23^ ameliorates liver fibrosis induced by HFD-plus-binge ethanol challenge, providing *in vivo* evidence that neutrophils promote liver fibrogenesis via the production of ROS. Although *p47*^phox^ is expressed predominantly in neutrophils, other cell types (eg, macrophages and so forth) also express p47^phox^ protein at lower levels.^23^ Therefore, it is plausible that p47^phox^ from other cell types also may contribute, to a lesser extent, to liver damage and fibrosis induced by HFD-plus-binge ethanol feeding.

Another important and unexpected finding from our study was that activated HSCs support the survival of neutrophils. Neutrophils have a short half-life of 6–8 hours and the liver is a major site where apoptotic neutrophils are removed via uptake by Kupffer cells.^28^ Spontaneous apoptosis is a key feature of neutrophils, and we also observed apoptotic neutrophils in the liver from HFD-plus-binge ethanol-fed mice. Because it was reported that hepatocyte apoptotic bodies can activate HSCs,^24^, ^25^ we tested the hypothesis that neutrophil apoptosis also may activate HSCs. However, our data showed that *in vitro* exposure to apoptotic neutrophils did not induce HSC activation but, instead, activated HSCs markedly promoted neutrophil survival. Furthermore, we provided 2 lines of evidence that the protective function of HSCs against neutrophil death is mediated via the production of GM-CSF and IL-15, which are 2 growth factors that play a critical role in sustaining the survival of neutrophils.^29^, ^30^, ^31^ First, highly purified HSCs produced high levels of GM-CSF and IL-15. Second, incubation with anti–GM-CSF or anti-IL-15 antibodies diminished the protective function of HSCs against neutrophil death. However, we could not rule out that HSCs also may produce other growth factors in addition to GM-CSF and IL-15 to promote neutrophil survival, and other cell types (eg, hepatocytes, macrophages, and so forth) also may produce survival factors for neutrophils *in vivo*. Interestingly, the genetic polymorphism I148M of the *Pnpla3* gene is strongly associated with steatohepatitis and fibrosis, and primary human I148M HSCs were shown to have significantly higher expression of GM-CSF.^32^ Thus, it is plausible to speculate that the association of the I148M *Pnpla3* gene in HSCs with liver fibrosis is mediated via the production of GM-CSF, which promotes neutrophil survival and activation. In addition, our present data suggest that HSCs also produce IL-15 and this cytokine promotes neutrophil survival. However, a recent study reported that IL-15 exerted a direct antifibrotic function by binding to IL15R on HSCs.^33^ Thus, IL-15 may play a dual role in the control of liver fibrogenesis via the direct inhibition of HSC activation and via the promotion of neutrophil survival.

The next obvious question is what the significance of HSC-mediated induction of neutrophil survival is. It is well known that spontaneous apoptosis of neutrophils ensures the termination of acute neutrophilic inflammation and transition to the resolution or chronic phase during organ damage.^34^, ^35^ One of the beneficial consequences of the prolonged survival of neutrophils is that they may help to clear the damaged hepatocytes during liver injury, but these neutrophils also may nonspecifically cause more hepatocyte damage and liver fibrosis via the production of ROS. In addition, it is known that the liver plays a key role in the activation of innate immunity against bacterial infection, and injured livers may become more susceptible to bacterial infection owing to the decreased antibacterial defense.^36^ Thus, prolonged survival of neutrophils induced by HSCs may help control bacterial infection during liver injury.

### Clinical and Therapeutic Implications

A clear clinical implication of our findings using the HFD-plus-binge ethanol feeding model is that obese individuals with severe fatty liver disease may be vulnerable to develop liver fibrosis, especially upon binge drinking. Patients with fatty liver diseases should be given sufficient education about the consequence of binge drinking and should be monitored more carefully. Our findings also suggest several therapeutic targets for the treatment of steatohepatitis. First, hepatic neutrophil infiltration mediated by CXCL1 is a critical step for both liver damage and liver fibrosis in the synergistic effect of obesity and alcohol binge drinking. High levels of hepatic CXCL1 and CXCL8/IL-8 expression also were reported in human nonalcohol steatohepatitis.^37^ Thus, CXCL1 and CXCL8/IL-8 may be potential therapeutic targets for steatohepatitis. Second, we found a close interplay between HSCs and neutrophils. Neutrophils induce hepatocyte damage but activate the HSCs via the production of ROS, thereby triggering liver fibrosis. Supporting the anti-oxidative capacity of hepatocytes may reduce liver damage and subsequently inhibit liver fibrosis. Finally, activated HSCs may promote the survival of neutrophils. Interfering with this effect by blocking the local function of GM-CSF and IL-15 may alleviate detrimental effects of neutrophils in inducing liver injury and inflammation. However, neutrophils also play an important role in suppressing bacterial infection and promoting liver regeneration.^38^ Recently, a clinical trial reported that treatment with G-CSF improved survival in patients with severe alcoholic hepatitis,^39^ which may be mediated via the G-CSF activation of neutrophils and subsequent inhibition of bacterial infection.^40^ Therefore, anti–GM-CSF treatment, which may be beneficial for the control of neutrophil overactivation, should be used with caution in patients with liver disease.
